# Endophytic Fungi as Potential Biocontrol Agents against *Sclerotium rolfsii* Sacc.—The Causal Agent of Peanut White Stem Rot Disease

**DOI:** 10.3390/cells11172643

**Published:** 2022-08-25

**Authors:** Mohammad Reza Safari Motlagh, Maryam Farokhzad, Behzad Kaviani, Dariusz Kulus

**Affiliations:** 1 Department of Plant Protection, Rasht Branch, Islamic Azad University, Rasht 4147654919, Iran; 2 Department of Horticultural Science, Rasht Branch, Islamic Azad University, Rasht 4147654919, Iran; 3 Laboratory of Ornamental Plants and Vegetable Crops, Faculty of Agriculture and Biotechnology, Bydgoszcz University of Science and Technology, Bernardyńska 6, 85-029 Bydgoszcz, Poland

**Keywords:** antagonist fungi, *Arachis hypogaea*, *Aspergillus flavus*, *Penicillium decaturense*, *Trichoderma virens*, *Trichoderma viride*

## Abstract

Peanut stem white rot caused by *Sclerotium rolfsii* Sacc. is a soil-borne disease that is widely prevailing across peanut farms, leading to serious economic losses. Screening for biocontrol agents against this pathogen is urgent. In this research, 166 fungal isolates including 136 isolates of *S. rolfsii* and 30 isolates of antagonistic endophytic fungi were obtained from a total of 220 samples collected from peanut farms in Guilan province, Iran. After morphological and molecular identification, six superior endophytic isolates were finally selected for the in vitro and greenhouse trials, including four isolates from *Trichoderma viride*, *Trichoderma virens*, *Penicillium decaturense*, and *Aspergillus flavus* and two isolates from *Penicillium rubens*. Four methods of biocontrol were used during the in vitro phase, i.e., dual culture, volatile metabolites assay, non-volatile metabolites assay (culture extract), and slide culture. It was found that *T. virens* had the highest capability of suppressing the mycelial growth of *S. rolfsii* in the dual culture method (90.98%). As for the volatile metabolites assay, the most effective isolates in inhibiting the pathogen’s mycelial growth were *P. rubens* (MN395854.1) and *A. flavus* (84.30% and 73.50% inhibition, respectively). In the non-volatile metabolites method, the isolates that performed the best in suppressing the mycelial growth of *S. rolfsii* were *T. viride* and *P. rubens* (MN395854.1) with 91.80% and 90.20% inhibitory effects, respectively. On the other hand, in the slide culture method, all isolates, except for *T. virens* and *T. viride*, successfully controlled the development of *S. rolfsii* hyphae. The greenhouse trials also supported the effectiveness of endophytic fungi in controlling the pathogen on the host plants. According to the results, *T. viride*, *A. flavus*, and *P. rubens* (MN395854.1) were 44%, 42%, and 38% effective in alleviating the disease incidence and severity. Moreover, the application of these antagonistic fungi in the greenhouse conditions increased the height, fresh weight, and dry weight of the *Arachis hypogaea* plants infected with the disease causal agent compared to the plants treated only with the pathogen. The results of the in vitro and greenhouse experiments revealed that the endophytic fungi occurring in the natural microbiota of peanut are capable of bio-controlling *S. rolfsii*, the causal agent of peanut stem white rot disease. These findings shed new insights into the possible resistance induction in *A. hypogaea* plants through biological protection.

## 1. Introduction

White stem rot disease, caused by *Sclerotium rolfsii* Sacc., is a major peanut (*Arachis hypogaea* L.) disease whose symptoms emerge as pale spots on the crown and dark brown spots on the underground part of the stems. First, the lower leaves and then the upper ones wilt quickly and start withering from up to down. The destruction of most xylem vessels causes the shoots to wilt [1]. *S. rolfsii* is one of the most serious pathogens invading peanut plants. This disease is responsible for 10–50% loss of the final peanut crop in South America, 27% loss of it in India, and over 25% loss in Australia. The loss rate in severely infected farms can even reach 80% [2]. Fungicide application is the strategy most used to control white stem rot disease. However, the intensive use of fungicides, especially of site-specific products, can select for resistant isolates (e.g., with a mutation at codon 240 causing resistance to a benzimidazole) and, consequently, may lead to control failures [3]. The genetic diversity of the causal fungus is the chief reason for the inefficiency of chemical and agronomic protection methods and the use of resistant peanut cultivars [4]. This reflects the significance of employing modern and environmentally friendly approaches to control plant diseases, such as biological control [5].

Upadhyay and Mukhyopadhayay [6] investigated the efficiency of *Trichoderma harzianum* Rifai against the sclerotia of *S. rolfsii* causing sugar stem rot disease (southern blight). The application of *T. harzianum* to the soil inoculated with the pathogen significantly reduced the severity of the sugar beet infection in the nursery. A research study on the biocontrol of the soybean crown and stem rot disease caused by *S. rolfsii* using *T. aureoviride* Rifai and the fungicide Tebuconazole revealed that the disease was less intense in the plants sown in the soil treated with the antagonist isolate. Moreover, the combined application of the bio-controlling factor and the fungicide in the greenhouse conditions increased the soybean yield and 1000-seed weight [7]. One should keep in mind, though, that *Trichoderma* spp. showed varied responses to different ambient temperatures and medium pH in suppressing the mycelial growth of *S. rolfsii* under in vitro conditions. Indeed, *T. harzianum* and *T. viride* Pers. were most effective in inhibiting the pathogenic mycelial growth at 37 °C and pH = 4 [8,9].

A study by Gancsan et al. [10] addressed the effect of simultaneously applied *T. harzianum* and some strains of the symbiotic bacterium *Rhizobium* spp. on *S. rolfsii*. The results showed that both the fungus and bacterium were effective in inhibiting the pathogen’s growth. As for the greenhouse conditions, the treatment of the plants with a combination of both alleviated the disease severity and improved the plant quality [10]. In a study on the biocontrol of *S. rolfsii*, which is also the causal agent of the southern blight of *Solanum lycopersicum* L., with *T. koningii* Oudem., the treatment of tomato seeds with the endophytic fungus and their subsequent sowing in pots reduced significantly the disease symptoms and the number of pathogenic sclerotia. Likewise, the treatment of tomato seeds with different *Trichoderma* isolates effectively reduced the collar rot disease intensity and increased the germination rate of the seeds [11]. Bosah et al. [12] compared the effectiveness of *Aspergillus*, *Penicillium*, and *Trichoderma* isolates in suppressing the growth of *S. rolfsii*. It was found that the *Trichoderma* spp. isolate had the strongest inhibitory effect on the pathogen growth. The isolates of *Aspergillus* spp. and *Penicillium* spp. were in the next ranks [12]. In a study on the control of white stem rot disease, Vikram and Hamzehzarghani [13] reported that seed treatment with an isolate of *T. harzianum*, strains of *Pseudomonas* *fluorescens* Migula, and the fungicide Captan performed fairly in controlling *S. rolfsii*. On the other hand, *T. viride* and *T. hamatum* (Bonord.) Bain. were effective in reducing the mycelial growth and the disease intensity of white stem rot of pepper, also caused by *S. rolfsii* [14]. In an in vitro dual culture experiment, various species of *Trichoderma* exhibited different results in suppressing the radial growth of mycelia and sclerotia of *S. rolfsii*. Among them, volatile metabolites of *T. viride* showed the greatest inhibitory effect. Similarly, *T. harzianum* and *T. viride* had the highest and *T. longibrachiatum* Rifai had the lowest effectiveness in inhibiting the radial growth of the pathogen mycelia in the dual-culture method [15]. In another study on the biocontrol of cucumber crown rot disease using five compost types, it was found that the unsterilized extract obtained from the compost containing the isolates of *Aspergillus* spp., *Cheatomium* spp., and *Penicillium* spp. had the highest inhibitory effect on the pathogen [16].

It has been reported that *A.* *niger* Tiegh. and *Penicillium* spp., as well as *Curvularia* sp., *Fusarium* sp., *T. harzianum*, *T. pseudokoningii* Rifai, *T. virens* (J.H. Miller, Giddens & A.A. Foster) Arx., and *T. viride* are inhibitory factors of the mycelial growth of *S. rolfsii* in vitro [17,18,19]. Doley et al. [20] found that the inoculation of groundnut seeds with symbiotic mycorrhiza fungi and *T. viride* was highly effective in stimulating the growth, expanding the nutrient uptake surface in the plant rhizosphere, and alleviating the damage of the white crown rot disease. Therefore, more research on the application of beneficial endophytic fungi in agriculture is needed, especially considering the environmental- and user-friendly aspect of the biological control compared to conventional plant protection [5].

This research aims to explore the performance of some isolates of antagonistic fungi collected from the natural microflora of *Arachis hypogaea*, in terms of their efficiency and efficacy in reducing the mycelial growth and disease intensity of white stem rot under in vitro and greenhouse conditions, and their effects on some morphological traits of the peanut plants.

## 2. Materials and Methods

### 2.1. Sampling, Isolation, Purification, and Identification of the Fungal Isolates

Samples were collected from the infected and uninfected *A. hypogea* plants in the main peanut-growing regions of Guilan province, Iran, including Astaneh-ye Ashrafiyeh, Kiashahr, Lasht-e Nesha, Talesh, Lisar, and Rezvanshahr, from June 2018 to August 2019. The samples collected from the infected parts of the plants (stems, crowns, leaves, fruits, and pods) were separately packed in plastic packages and sent to the laboratory to be stored at 4–5 °C. To isolate *S. rolfsii*, 2–5 mm fragments were cut from the space between the infected and uninfected tissues of the leaves, stems, fruits, and pods. Surface disinfection was performed with 5% (*v/v*) sodium hypochlorite (NaOCl) for 1 min, followed by three times of surface rinsing with sterile distilled water for 30 s. After draining, the fragments were placed in a potato dextrose agar (PDA) (Merck Millipore, Burlington, MA, USA) medium in Petri dishes and incubated at 25 °C for three days [21]. The isolates were purified with the hyphal tip method. Then, the Petri dishes were kept at 27 °C. The fungal sclerotia formed after 7–10 days of culture were collected and drained on filter paper. Next, they were put in sterile microtubes and stored at −20 °C [22].

To isolate the studied antagonistic isolates, 5–10 mm fragments were sampled from the healthy tissues of leaves, stems, crowns, and fruits. Next, they were rinsed with sterile distilled water for 30 s. After draining, the samples were transferred to PDA-containing Petri dishes in a sterile hood. After incubation at 26 °C for 2–3 days, the fungal colonies were isolated and a disk from each colony was transferred to another Petri dish that contained the PDA culture medium. Following incubation for another 2–3 days, a patch of the fungal colony was put in the 1.5% (*w/v*) water agar (WA) culture medium [21]. Next, after 2–3 weeks and spore production, the fungi were purified with the single spore method. Diagonal test tubes containing the PDA medium were used to keep the fungal isolates.

After isolation and purification, the fungi were identified based on their macroscopic morphological features, such as colony shape and color and the growth type in the PDA medium. Then, their microscopic characteristics were studied (including the mycelia growth, singularity or plurality of conidiophores, their color and dimensions, length, width, and the number of septa, as well as the shape and size of conidia in the WA culture medium) by preparing microscopic slides and using valid keys [23,24,25,26].

### 2.2. Genetic Analysis

The fungal isolates were cultured in the PDA medium. At the end of the incubation period and after the full growth of the samples, their genomic DNAs were extracted using the extraction kit of CinnaGen (Tehran, Iran) as per the company’s procedure.

For the molecular identification of the fungal isolates, the genomic ITS-rDNA region was assessed and the polymerase chain reaction (PCR) was performed with a T-100 thermocycler (Bio-Rad, Hercules, CA, USA) to amplify the ITS-rDNA region [27]. The direct and reverse primer pairs required for the reaction were designed using the NCBI gene database and the Primer3 software package [28] and were then synthesized by Sinaclon, Tehran, Iran (Table 1).

The initial PCR reaction was performed at a final volume of 25 µL using a PCR master mix by CinnaGen. The PCR thermal profile was set following White et al. [27] for amplifying the ITS-rDNA genomic region, and the electrophoresis was performed in the 1.5% (*w/v*) agarose gel (Sigma-Aldrich, St. Louis, MO, USA) at 120 V for 2 h. The electrophoresis was re-checked. The PCR temperature program included 94 °C for 5 min, followed by 35 cycles of 94 °C for 30 s, gradient temperature of 53–58 °C for 30 s, 72 °C for 30 s, and 72 °C for 7 min.

The PCR products of the studied samples were sequenced by Sinaclone (Tehran, Iran), and the similarity of the target sequence with the available sequences was assessed by the NCBI database using the BLAST algorithm [29]. The data from the sequence similarity analysis were phylogenetically assayed by the neighbor-joining (NJ) method.

### 2.3. In Vivo Pathogenicity Test

The pathogenicity test of the 166 detected fungal isolates was assayed. For this purpose, plastic pots with a diameter of 15 cm were filled with garden soil and compost (1:1) sterilized in an autoclave. Then, the seeds of *A. hypogaea* cultivar ‘NC2’ were disinfected with 30% (*v/v*) NaOCl for 1 h. The inoculum of the antagonistic fungi was prepared at a rate of 4 × 10^6^ spores per 1 mL of sterile distilled water for each fungus after adding Tween-20 at a concentration of 1% (*v/v*). Next, the seeds were artificially inoculated. In each pot, a germinated (72 h after placing on wet filter paper) and treated (inoculated with antagonistic fungi) peanut seed was sown at a depth of 3–5 cm of the sterile substrate and then, the pots were watered and put in a greenhouse in August, in natural light conditions [28]. In each pot, about 10–20 sclerotia were placed in the vicinity of each peanut seed at a depth of 2 cm [30]. The visual symptoms of the disease were recorded daily from the second week of sowing to three months, i.e., mid-November concurrent with the pegging phase and then, the formation of the first pods. The greenhouse conditions were set at 27 °C with a saturated humidity of over 90% and the plants were irrigated as required [31]. Finally, 6 fungal isolates were selected as antagonistic isolates from the plants that exhibited no disease symptoms. As for the *S. rolfsii* pathogen, the isolate with the most extensive macroscopic symptoms and the highest disease intensity was selected for the in vitro biocontrol assays.

### 2.4. In Vitro Biocontrol Assay

#### 2.4.1. Dual Culture Method

In this method, a 5 mm mycelial disc, which was taken from the margins of a 5–7-day-old culture of *S. rolfsii*, was placed in an 8 cm PDA-containing Petri dish at a distance of 2 cm from the dish edge under a sterile hood. After 48 h in an incubator at 26 °C, a 5 mm mycelial disc, which was taken from the margins of the 5–7-day-old culture of antagonist fungi, was placed 3 cm away from the pathogenic fungus. The Petri dishes were stored at 26 °C and the mycelial growth of the fungus was measured seven days later. In the control dishes, a mycelial disc from the margins of the 5–7-day-old culture of *S. rolfsii* was placed at the center of a Petri dish, in sterile conditions, and stored in conditions similar to the other treatments [32]. At the end of the storage period, the radial growth of *S. rolfsii* was measured in the control and treatment. The reduction in the radial growth versus the control was measured by

$$\mathrm{Mycelial}\;\mathrm{growth}\;\mathrm{inhibition}\;\left(\%\right)=\frac{C\;-\;T}{C}\times \;100$$

where *C* denotes the radial growth of *S. rolfsii* in the control Petri dishes and *T* denotes the radial growth of the pathogen in the presence of the studied fungi [32].

#### 2.4.2. Assay of Volatile Metabolites Effect

In this assay, 5 mm discs of the pathogen and each antagonistic isolate used in the experiment were separately cultured on the PDA medium in 8 cm Petri dishes. The Petri dishes were decapped in sterile conditions, and their bottoms, which contained the culture medium and the colonies of the pathogen and antagonistic fungi, were placed in front of each other and enclosed with parafilm. In the control sample, a Petri dish containing the fungi-free PDA culture medium was placed in front of the Petri dish containing *S. rolfsii*. After seven days of incubation at 30 °C, i.e., until the colony of the control sample grew to the brim of the Petri dish, the radial growth of the fungi in the Petri dishes was recorded, and the inhibition of the pathogen growth by different fungal isolates was calculated [33].

#### 2.4.3. Assay of Growth Inhibitory Effect of Non-Volatile Compounds (Culture Extract)

Isolates of the studied fungi were cultured in 250 mL Erlenmeyer flasks containing 150 mL of the potato dextrose broth (PDB) culture medium [34] and were shaken at a rate of 70 rpm at 26 °C for 10 days. They were, then, extracted using biological filters with a pore diameter of 0.22 µm (Merck Millipore, Burlington, MA, USA) and a vacuum pump. The filtered extract with 25% dilution was added to the PDA culture medium. The extract that was added to the PDA culture medium in the control Petri dishes contained no fungi. A mycelial disk from the 3-day-old culture of *S. rolfsii* was placed at the center of the treatment and control Petri dishes. After seven days of storage at 26 °C, the radial growth of the pathogen was measured in the control and treatment [35].

#### 2.4.4. Hyperparasitism Test (Slide Culture)

A clean slide was first placed on two L-shaped glass bars inside a 12 cm Petri dish and sterilized. Then, 2% (*w/v*) molten WA culture medium was poured onto the slide to form a thin layer of agar on it. In the next step, small mycelial discs of the target antagonistic fungus and *S. rolfsii* were placed on slides at a distance of 2 cm from one another. Then, the Petri dishes were stored at 26 °C. After seven days, features of fungal hyphae that reached one another and their interactions with each other were investigated using an optic microscope [32].

### 2.5. Biocontrol Studies in the Greenhouse

First, the seed biopriming method was used, for which 3–5 mm discs of the target antagonistic isolates were first cultured in Erlenmeyer flasks containing 150 mL of the PDB culture medium and shaken at 120 rpm at 28 °C for 10 days. When the vegetative growth of the fungus was maximized, the inoculums of the antagonistic fungi were prepared by using a hemocytometer to prepare spore suspensions containing 10^8^ spores per 1 mL of sterile distilled water for the isolates of *Trichoderma* and *Penicillium* and 10^6^ spores per 1 mL for the *Aspergillus* isolate, respectively. The inoculums were used for the artificial inoculation of the *A. hypogaea* ‘NC2’ seeds. The seeds were disinfected in 30% (*v/v*) NaOCl for 1 h and were rinsed with sterile distilled water thrice. To induce germination, seeds were placed in a damp environment at 25 ± 2 °C for 72 h. Next, 3–4 seeds were put on a shaker in an Erlenmeyer flask containing the target fungal spore suspension (for 30 min) for their inoculation. The *S. rolfsii* inoculum was prepared by the sclerotium production and propagation method [36]. In this method, 10 mL of the PDA culture medium (39 g·L^−1^) was poured into Petri dishes and inoculated with 5 mm discs from the 3-day-old culture of *S. rolfsii*. The cultures were then incubated at 25 °C for three weeks, after which the number and size of sclerotia produced by the pathogen were counted. Then, 130 plastic pots with a diameter of 15 cm were filled with garden soil and compost at equal ratios. The pots were sown each with one germinated and one treated peanut seed, watered, and placed inside a greenhouse. The greenhouse temperature was set at 27–31 °C with 50–70% humidity throughout the study, and the irrigation was performed regularly [28]. Six to ten days after the treated seeds were sown, about 10–20 *S. rolfsii* sclerotia were put in the soil at a depth of 2 cm in the vicinity of the peanut seeds in all treatment pots [30].

A completely randomized design with nine treatments at four levels and three replications was used to measure the severity, incidence, and severity alleviation of the disease. The morphological traits of the plants, including their height, fresh weight, and dry weight, were measured to investigate the effects of six antagonistic fungi on *S. rolfsii.* Moreover, three control groups were included, i.e., (1) plants inoculated with distilled water alone, (2) plants inoculated with *S. rolfsii* alone, and (3) plants inoculated with antagonistic fungi alone. The visual changes and symptoms induced by *S. rolfsii* were recorded daily from the second week of sowing to three months, i.e., mid-November [31]. The disease severity was measured by Horsfall and Barratt’s [37] scale with slight modifications. The disease severity for each plant was determined using the disease severity index [38] and then, the disease severity was quantified by:

$$\mathrm{Disease}\;\mathrm{severity}=\frac{\;(N_{1}\;\times \;1)+(N_{2}\;\times \;2)+\ldots \ldots \left(N_{t}\;\times \;t\right)}{N_{1}+N_{2}+\ldots \;N_{t}}$$

where *N* represents the number of crowns (stems) at each degree of disease severity [38].

The disease incidence or infection percentage, which shows the number of the infected plants and stems (crowns) per total number of plants and stems, was estimated by

$$I=\sum^{\;}\frac{X}{N}$$

where *I* represents the extent of disease incidence, *X* represents the number of infected plants and stems or crowns, and *N* represents the total number of the evaluated plants [39].

### 2.6. Statistical Analysis

Data collected from the in vitro assays and greenhouse studies were statistically analyzed using the SAS software package. The analysis of variance (ANOVA) and the comparison of mean data were conducted by the least significant difference (LSD) test. Three replications were considered in all the experiments. The experiments were repeated at least twice.

## 3. Results

### 3.1. Taxonomy of Fungal Isolates Based on Morphological and Molecular Traits

After the initial identification, out of the 166 fungal isolates at the genus level, 6 isolates that did not cause peanut disease in the pathogenicity test in vivo were selected for the biocontrol and greenhouse assays. These isolates were identified at the species level using morphological and molecular methods.

**Figure 1:**
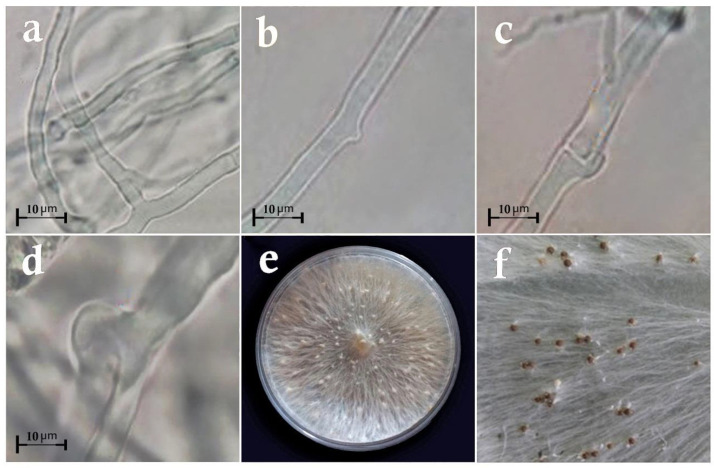
Microscopic images of *Sclerotium rolfsii*: (**a**,**b**) mycelia; (**c**,**d**) clamp connection; (**e**) mycelium distribution pattern on the PDA medium; (**f**) sclerotia formation.

**Figure 2:**
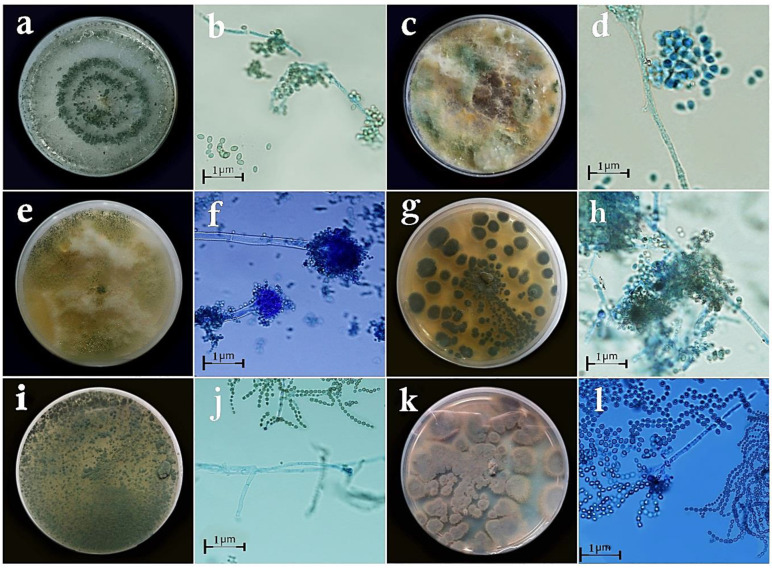
Antagonistic fungi used in the study: (**a**) colony of *Trichoderma viride* on PDA medium; (**b**) conidia and conidiophores of *Trichoderma viride*; (**c**) colony of *Trichoderma virens* on PDA; (**d**) conidia and conidiophores of *Trichoderma virens*; (**e**) colony of *Aspergillus flavus* on PDA; (**f**) conidia and conidiophores of *Aspergillus flavus*; (**g**) colony of *Penicillium rubens* (MN395854.1) on PDA; (**h**) conidia and conidiophores of *Penicillium rubens* (MN395854.1); (**i**) colony of *Penicillium rubens* (MN395851.1) on PDA; (**j**) conidia and conidiophores of *Penicillium rubens* (MN395851.1); (**k**) colony of *Penicillium decaturense* on PDA; (**l**) conidia and conidiophores of *Penicillium decaturense*.

**Figure 3:**
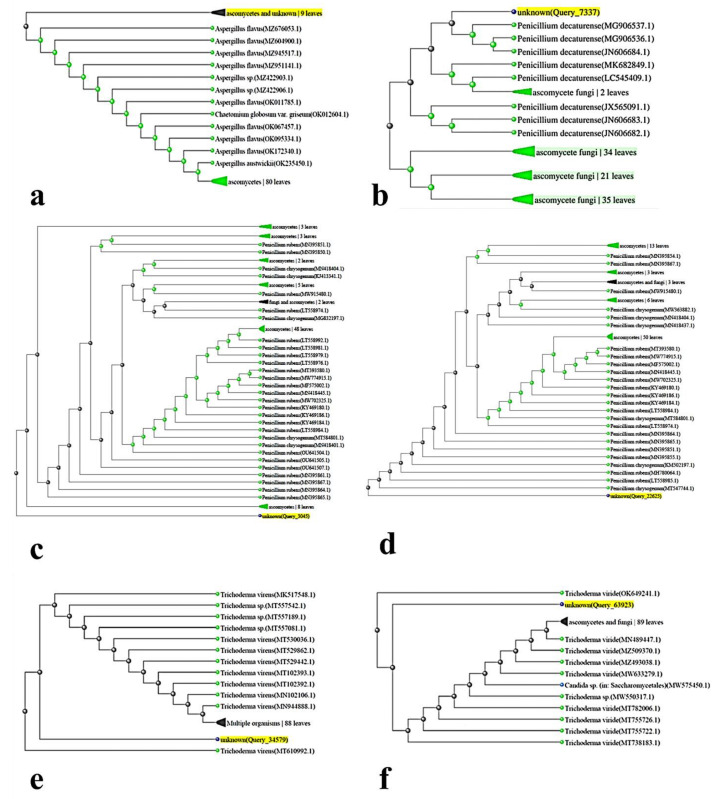
Phylogenetic trees resulting from the ITS-rDNA sequence analysis by the neighbor-joining method represent the location of the searched isolate in the vicinity of the cluster related to: (**a**) *Aspergillus flavus*, (**b**) *Penicillium decaturense*, (**c**) *Penicillium rubens* (MN395851.1), (**d**) *Penicillium rubens* (MN395854.1), (**e**) *Trichoderma virens*, (**f**) *Trichoderma viride*.

The results of studying the antagonistic fungal isolates by comparing morphological traits, checking the similarity of DNA sequences in the NCBI database, and drawing phylogenetic trees by the neighbor-joining method revealed that two isolates were related to *Penicillium rubens* Biourge and four other isolates to *Penicillium decaturense* S.W. Peterson, *Aspergillus flavus* Link E.M. Bayer & Wicklow, *Trichoderma virens* (J.H. Miller, Giddens & A.A. Foster), and *Trichoderma viride* Pers. On the other hand, the pathogenic fungal isolate belonged to the species *Sclerotium rolfsii* Sacc. (Figure 1, Figure 2 and Figure 3).

### 3.2. In Vitro Biocontrol Assays

**Figure 4:**
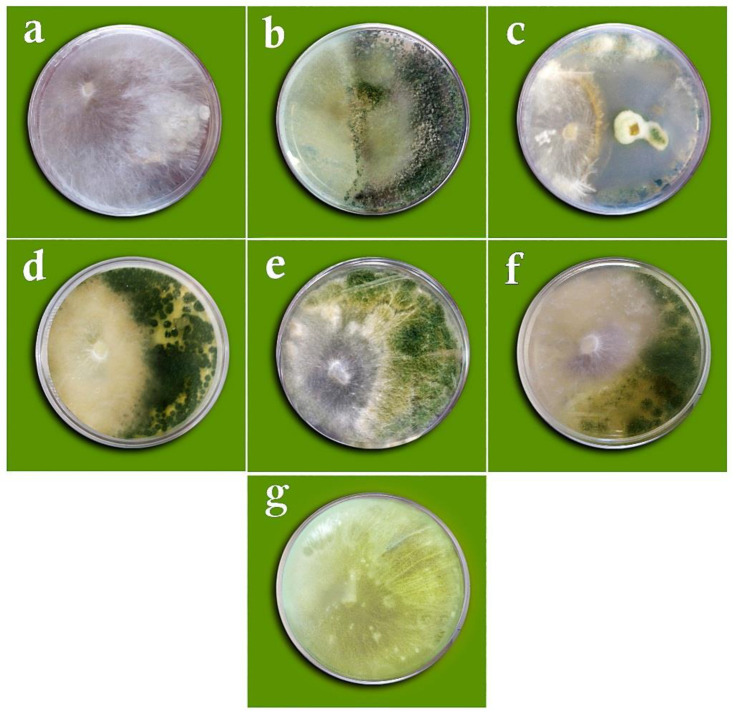
Dual culture test of *Sclerotium rolfsii* versus the studied fungi on the PDA medium: (**a**) *Sclerotium rolfsii* only (control), (**b**) *Trichoderma viride*, (**c**) *Trichoderma virens*, (**d**) *Penicillium rubens* (MN395854.1), (**e**) *Aspergillus flavus*, (**f**) *Penicillium decaturense*, (**g**) *Penicillium rubens* (MN395851.1).

**Figure 5:**
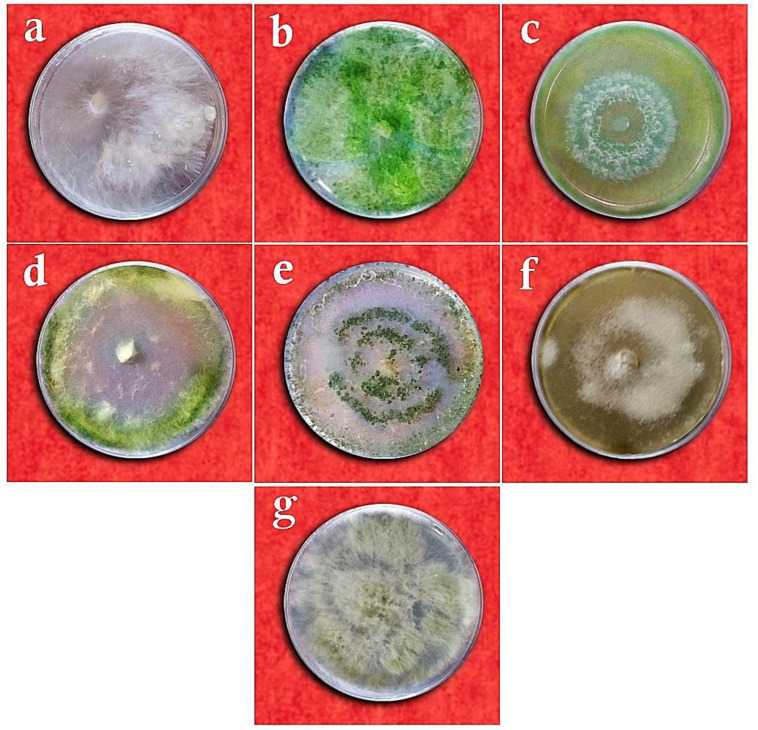
Volatile metabolites test of *Sclerotium rolfsii* versus the studied fungi on the PDA medium: (**a**) *Sclerotium rolfsii* (control), (**b**) *Penicillium rubens* (MN395854.1), (**c**) *Aspergillus flavus*, (**d**) *Penicillium rubens* (MN395851.1), (**e**) *Trichoderma viride*, (**f**) *Trichoderma virens*, (**g**) *Penicillium decaturense*.

**Figure 6:**
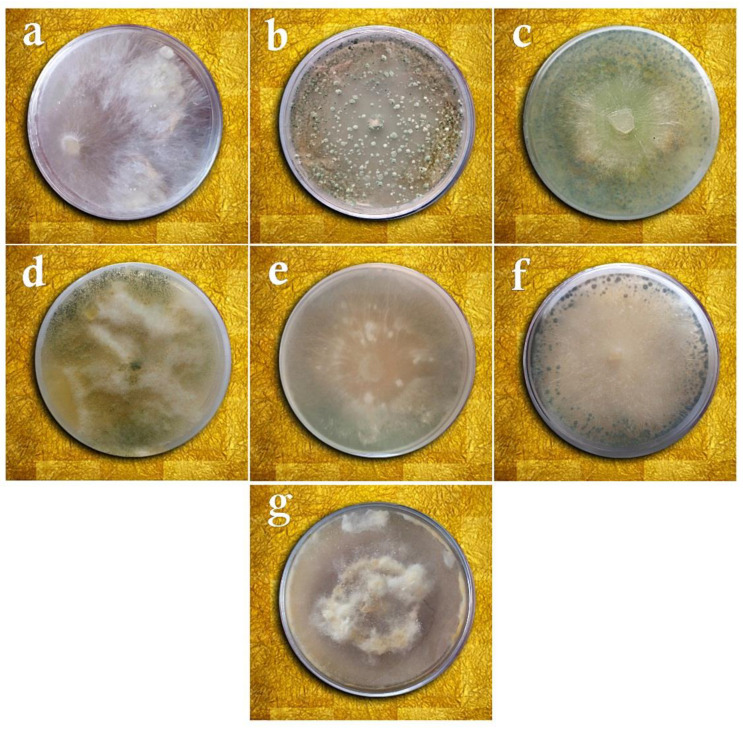
Non-volatile metabolites test of *Sclerotium rolfsii* versus the studied fungi on the PDA medium: (**a**) *Sclerotium rolfsii* (control), (**b**) *Trichoderma viride*, (**c**) *Penicillium rubens* (MN395854.1), (**d**) *Aspergillus flavus*, (**e**) *Penicillium decaturense*, (**f**) *Penicillium rubens* (MN395851.1), (**g**) *Trichoderma virens*.

According to the results of the ANOVA, the studied treatments differed significantly in terms of the mycelial growth inhibition of *Sclerotium rolfsii* in the dual culture, volatile metabolites, and non-volatile metabolites assays (*p* ≤ 0.01). The comparison of means for the mycelial growth inhibition in the dual culture method by the LSD test revealed that the *T. virens* isolates were most capable (90.98%) of inhibiting the growth of *S. rolfsii*, whereas a nil inhibition percentage was reported for *P. decaturense* and *P. rubens* (MN395851.1), which differed significantly from the other fungal species (Table 2 and Figure 4). In the volatile metabolites test, *P. rubens* (MN395854.1) showed the highest capability in inhibiting the mycelial growth of *S. rolfsii* (84.30%) while *P. decturense*, *T. viride*, and *T. virens* showed the lowest (0%) growth inhibition percentage (Table 2 and Figure 5). In the non-volatile metabolites test, the highest mycelial growth inhibition was reported with *T. viride* and *P. rubens* (MN395854.1). No growth inhibition was found with the other four antagonistic fungi species (Table 2 and Figure 6). In the above three methods, the antagonistic isolates that effectively reduced the mycelium growth of the pathogen also completely prevented the formation of sclerotia, and the isolates that were not effective in inhibiting the pathogen’s growth reduced the number of sclerotia by 40%.

**Figure 7:**
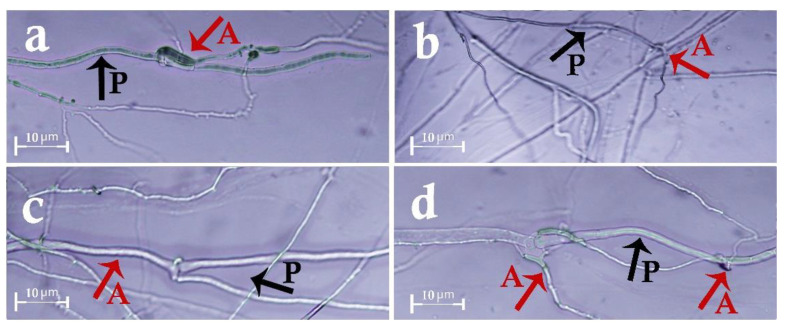
Hyphal coiling and hyperparasitism of the studied fungi on *Sclerotium rolfsii*: (**a**) *Penicillium decaturense,* (**b**) *Aspergillus flavus*, (**c**) *Penicillium rubens* (MN395851.1), (**d**) *Penicillium rubens* (MN395854.1), magnification 460×, arrows point to antagonistic fungi (A) and pathogen (P).

In the slide-culture method, the hyphae of *P. decaturense* reached the hyphae of the pathogen, enclosed them, especially at the crossing points, and created hooks that cut the hyphae of *S. rolfsii* and caused their collapse (Figure 7a). The hyphae of *A. flavus* reached the hyphae of *S. rolfsii*, enclosed them, and sometimes penetrated the pathogen hyphae by clip-shaped links. Then, they created coil-shaped impediments and stopped the hyphae growth of *S. rolfsii*, destructed their walls, and, in some cases, parasitized them by generating plenty of spores (Figure 7b). On the other hand, the hyphae of *P. rubens* (MN395851.1) progressed along the pathogen hyphae and, in some cases, penetrated it by creating hooks and rings that stopped the growth of *S. rolfsii* and destructed its cell walls by spiral movement (Figure 7c). In some cases, *P. rubens* (MN395851.1) produced a lot of spores, thereby parasitizing the pathogen hyphae. The hyphae of *P. rubens* (MN395854.1) and pathogen reached one another. Then, the antagonist bonded to the wall of *S. rolfsii* hyphae by creating hook-shaped branches and induced changes in the structure of the pathogen hyphae by penetrating them (Figure 7d). The hyphae of *T. viride* and the pathogen did not reach each other, so they had no interaction. Similarly, no interaction was observed between *T. virens* and the *S. rolfsii* pathogen.

### 3.3. Biocontrol Studies in Greenhouse Conditions

**Figure 8:**
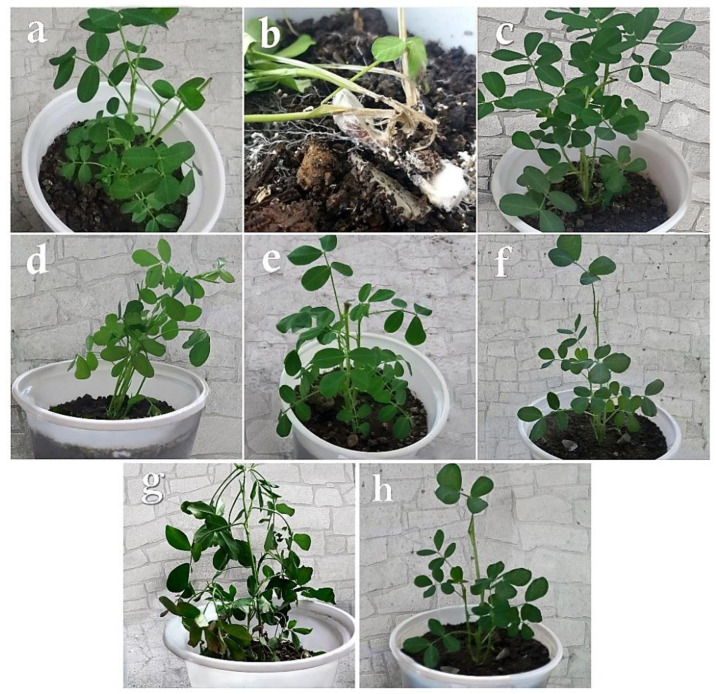
Greenhouse cultivation of *A. hypogaea* plants: (**a**) control plants treated with distilled water, (**b**) symptoms of the disease on the plants after inoculation only with *Sclerotium rolfsii*, (**c**) inoculation only with *Trichoderma viride*, (**d**) only with *Aspergillus flavus*, (**e**) only with *Penicillium rubens* (MN395854.1), (**f**) only with *Penicillium rubens* (MN395851.1), (**g**) only with *Penicillium decaturense*, (**h**) only with *Trichoderma virens*.

In the control plants inoculated with distilled water (Figure 8a) or any of the antagonist fungi alone (Figure 8c–h), no disease symptoms were observed. In the control plants treated solely with *S. rolfsii*, the first disease symptoms appeared 10–14 days after the inoculation. The pathogen hyphae infected the plants completely so that the plants withered visually and were destroyed at the crown region in addition to lesions on their crowns and stems. With the progress of the disease symptoms over 21 days, the plants were destroyed and a lot of sclerotia were formed on the residues of the dead peanuts (Figure 8b).

**Figure 9:**
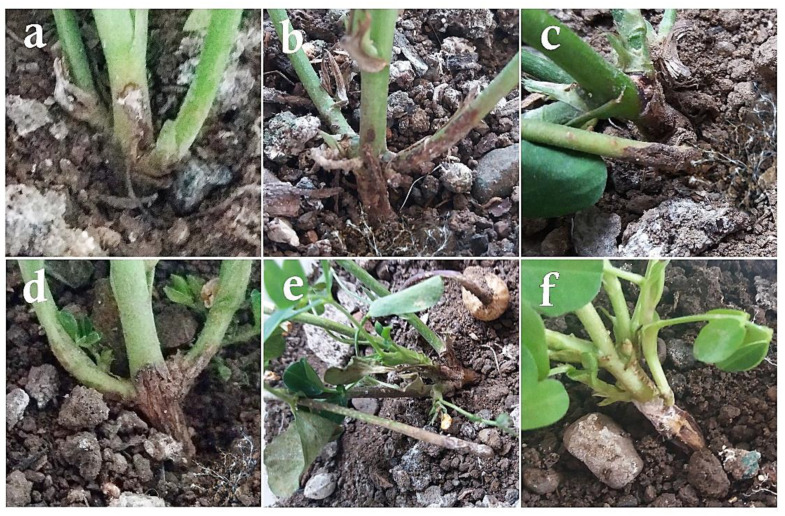
Greenhouse cultivation of *A. hypogaea* plants: (**a**) symptoms of the disease on the plants after inoculation with *Sclerotium rolfsii* and *Trichoderma viride*, (**b**) after inoculation with *Sclerotium rolfsii* and *Aspergillus flavus*, (**c**) after inoculation with *Sclerotium rolfsii* and *Penicillium rubens* (MN395854.1), (**d**) after inoculation with *Sclerotium rolfsii* and *Penicillium rubens* (MN395851.1), (**e**) after inoculation with *Sclerotium rolfsii* and *Penicillium decaturense*, (**f**) after inoculation with *Sclerotium rolfsii* and *Trichoderma virens*.

In the plants treated with *T. viride* and the pathogen, the disease symptoms appeared 14–20 days after the inoculation in the form of pathogen hyphae growth at the soil surface and around the plant crown. The pathogen hyphae covered the plant stem and created pale spots at the infection place. As the plant aged, the spots became lesions and caused the local withering of the infected plant stem (Figure 9a). The disease symptoms in the plants treated with *A. flavus* and *S. rolfsii* appeared 10–15 days after the inoculation as the growth of the pathogen hyphae around the plant crown. Gradually, the pathogen hyphae penetrated the plant stem and created pale spots on them, which became lesions around the stem (Figure 9b). In the plants treated with *P. rubens* (MN395854.1) and *S. rolfsii*, the first disease symptoms appeared 14–16 days after the inoculation as the growth of hyphae around the plant crown and pale spots in this region. Then, as the disease expanded around the stem, the spots gradually became lesions (Figure 9c). In the plants treated with *P. rubens* (MN395851.1) and the pathogen, the first symptoms were observed 16–20 days after the inoculation in the form of fungal hyphae growth at the soil surface and around the plant crown. Gradually, the hyphae progressed toward the plant crown, and as the infection penetrated the stem tissue and expanded, relatively large lesions were created around the central stem. Lesions of fair sizes were also observed on the adjacent stems. As the symptoms of the disease progressed, sclerotization could also be seen in the remains of the diseased stems (Figure 9d). The symptoms appeared 16 days after inoculation in the plants treated with *P. decaturense* and the pathogen in the form of pathogen hyphae growth at the soil surface and around the plant crown. The spots become darker as they progressed toward the stem. They become lesions first on the central stem and then on the adjacent stems (Figure 9e). In the plants treated with *T. virens* and the pathogen, the first symptoms were observed 16–20 days after the inoculation as the growth of fungal hyphae at the soil surface and around the plant crown. As the disease symptoms developed, the whole stems gradually became infected. A relatively big lesion was created on the central crown as it covered 70–90% of the crown and the adjacent crowns were weakened and showed local necrosis. Sclerotization could also be seen in the remains of the diseased stems (Figure 9f).

ANOVA revealed significant differences among the studied treatments in terms of disease severity at the *p* ≤ 0.05 level (Table 3). Based on the results of the LSD test, the lowest disease severities were 2.80 for the treatment of *T. viride* and 2.90 for the treatment of *A. flavus*. On the other hand, the highest disease severities were related to *S. rolfsii* (only) and *T. virens*. Accordingly, the treatments of *T. viride*, *A. flavus*, and *P. rubens* (MN395854.1) were effective in suppressing the severity of the disease caused by *S. rolfsii*. The treatments of *P. decaturense* and *P. rubens* (MN395851.1) were in the next ranks (Table 3). There were also significant differences among the treatments in terms of the disease incidence by *S. rolfsii*. The LSD test showed that the lowest incidence levels were 31% for the treatment with *T. viride* and 32% for *A. flavus*, but they did not differ significantly from the treatment with *P. decaturense* (38%). The highest incidence (50–52%) was related to the treatments with *T. virens* and *P. rubens* (MN395854.1) (Table 3).

The results of ANOVA for the morphological traits showed that the studied treatments affected the plant height and fresh weight significantly at the *p* ≤ 0.01 and *p* ≤ 0.05 levels, respectively (Table 4). The highest plants were obtained in the treatments of *T. viride* *+* *S.* *rolfsii* and *P. rubens* (MN395854.1) *+* *S.* *rolfsii*, while the lowest from the treatment of *S. rolfsii* only. The latter plants were approximately two-fold shorter than all other experimental objects. The highest plant fresh weight was found after the treatment with *T. viride* *+* *S.* *rolfsii*, while the lowest with the treatment of *T. virens* (only) and *S.* *rolfsii* (only), differing significantly from all other treatments (Table 4). As for the plant dry weight (*p* ≤ 0.01), it was found that the highest shoot + root DW was obtained from the treatment of *P. decaturense* + *S.* *rolfsii*, which differed from the other treatments significantly. The lowest DW was related to the treatment with *S.* *rolfsii* only (Table 4).

## 4. Discussion

Plant pathogens cause losses of approximately 16% globally. Hence, management measures must be implemented to mitigate losses and guarantee safe and effective food production, for example, through the application of induced resistance, biological control, and endophytes [5].

In this research, when the dual culture method was used, *T. virens* exhibited the highest inhibitory effect against *S. rolfsii*. On the other hand, *T. viride*, *P. rubens* (MN395854.1), and *A. flavus* exhibited a good to moderate inhibitory effect, whereas *P. decaturense* had the lowest inhibitory effect on the mycelial growth of the pathogen. Since *S. rolfsii* had a faster growth rate on the PDA medium in the dual culture method, the studied antagonistic fungi might have been more successful in the competition over nutrients and space. Moreover, the synthesis of antibiotics and semi-antibiotics by the fungi could determine the efficiency of the studied isolates. This is consistent with the research of Basumatary et al. [17] on the efficiency of *T. viride* and *Penicillium* sp. isolates in suppressing the mycelial growth of *S. rolfsii* in the dual culture. Likewise, in the present study, the isolates of *T. viride*, *Penicillium* sp., and *A. flavus* had high inhibitory effects on the development of the pathogen, which coincides with some other studies [40,41].

In the volatile metabolites assay, *P. rubens* (MN395854.1) was the most efficient, and the isolates of *P. decaturense*, *T. virens*, and *T. viride* were the least effective in suppressing the mycelial growth of *S. rolfsii*. Better mycelial growth inhibition by *P. rubens* (MN395854.1) versus the other studied isolates can be attributed to the production of more volatile compounds by this isolate, such as 6-pentyl-α-pyrone, which is consistent with another study on the effect of *Trichoderma* isolates and their culture method on the quantity and quality of volatile metabolites produced [42,43,44,45]. In the study by Rekha et al. [46], various *Trichoderma* species have differed in their capability of suppressing mycelial growth and the production of components constituting *S. rolfsii* sclerotia in different in vitro assays, such as volatile metabolites, which agrees with the in vitro observations of the *Trichoderma* species in the present study.

The present research showed that the non-volatile compounds produced by the antagonistic fungi were more effective in suppressing the mycelial growth of *S. rolfsii* than the volatile chemicals, which can be associated with the fact that pathogenic fungi differ in their sensitivity to biochemically different compounds [47]. The various effects of different antagonistic isolates on suppressing the mycelial growth of *S. rolfsii* can be related to their genetic characteristics, the host plant, and the sites where they were collected [48]. The isolates of *T. virens* and *T. viride* have different acting mechanisms, such as saprophytic, competition, antibiosis, mycoparasitism, induced resistance, and soil bioremediation, and act as an opportunist and non-pathogenic symbiotics in plants and induce resistance to biotic and abiotic factors in some cases [49]. As such, the isolate of *T. viride* has probably used the mechanism of competition over food, induced resistance, and the establishment of symbiotic relationships with plant roots to alleviate disease severity in *A. hypogaea* cultivated in the greenhouse.

In the slide culture method, the hyphae of the studied antagonistic isolates (except *T. virens* and *T. viride*) had a positive tropism toward the cell wall of the pathogen hyphae due to the presence of chemicals such as lectin, so their hyphae progressed along with the pathogen and, in some cases, penetrated its hyphae by creating appressorium, ring-shaped, and/or hook-shaped organs. Accordingly, *P. decaturense* isolate had the highest, and *T. virens* and *T. viride* isolates had the lowest efficiency in hyperparasitism and the destruction of the pathogen hyphae. Another research showed that *T. harzianum* decomposed the cell wall of the pathogen since its hyphae coiled around *S. rolfsii* and formed a sucking organ inside of it [8]. It has been shown that *T. harzianum* disintegrates the hyphae of *S. rolfsii* by secreting specific hydrolyzing enzymes in response to biochemical signals synthesized by the pathogen [49,50].

Interestingly, previous research introduced *A. flavus* as the causal agent of the peanut kernel rot [51], whereas the isolate studied in this paper was found to be non-pathogenic and exhibited good to moderate efficiency in the greenhouse and in vitro conditions for the suppression of *S. rolfsii*. This inconsistency in the results can be related to the difference in the studied isolates’ pathogenicity. *Aspergillus flavus* is a common soil fungus. The major concern with this species is that it produces carcinogenic toxins called aflatoxins, which are associated with food and feed safety hazards [26]. Therefore, caution should be taken in recommending this isolate as an antagonist agent.

The greenhouse tests in the present study using seed bio-priming, in which the seeds were primed with beneficial and biological factors, such as *Trichoderma*, instead of chemicals [52], revealed that the plants treated with *T. viride* were most capable of reducing the disease severity of *S. rolfsii*. Since the peanut seeds treated with the isolates of *Trichoderma* were sown about one week before soil inoculation with the disease agent, the fungi used in the biological control may have had enough chance of deployment in the soil by using specific mechanisms, such as the secretion of enzymes and antibiotics.

The presence and abundance of *Trichoderma* spp. in the soil environment, competition over nutrients in the plant rhizosphere, and competition over seed secretions are significantly influential in controlling the disease severity and increasing plant height, fresh weight, and dry weight. In Latunda’s [53] study, when commercial *T. koningii* powder was added to the potting soil inoculated with *S. rolfsii*, not only were the symptoms of tomato south blight controlled significantly, but the production of disease-causing sclerotia was also decreased remarkably. Similarly, the treatment of tomato seeds with the TR05, TR06, and TR08 isolates of *Trichoderma* had similar effects in Islam et al. [11]. In the present study, the application of *Trichoderma* isolates, e.g., *T. viride*, increased the growth rate and plant emergence uniformity in stressful conditions, which over the years agrees with the results of Parera and Cantliffe [54]. This highlights the fact that, at least, some endophytic fungi still maintain their efficiency in controlling the growth of pathogenic fungi on farms.

Greenhouse and field trials proved the positive effects of *T. harzianum* and *Talaromyes flavus* C.R. Benj. in bio-suppressing garlic white rot caused by *S. cepivorum* and their stimulating impact on plant vegetative parameters, including plant height and its yield [55]. Similarly, we observed increases in peanut plant height, fresh weight, and dry weight after the isolates of *T. viride* and *A. flavus* were applied. The fungi *Aspergillus* sp., *Penicillium citrinum* Thom, and *P.* *spirillum* Pitt induce the production of secondary alkaloid metabolites in plants [56]. These compounds are involved in plant protection from microbial pathogens, plant competition with one another, and plant symbiosis with microbes [57]. It seems that the fungi studied in this research used similar mechanisms after deployment on the plant and induced physico-chemical changes in its structure, thereby exhibiting similar performance in suppressing the disease agent and optimizing the plant’s morphological traits. In Sabet et al. [16], decreases in the severity and incidence of cucumber crown rot disease and increases in plant height, root length, and volume, as well as plant fresh and dry weight were recorded in the plants inoculated with *Penicillium* and *Cheatomium* along with *S. rolfsii*. Likewise, the application of the mycelial powder of *P. chrysogenum* Thom as an effective bio-controlling factor has been reported to mitigate the damages of *Fusarium* and *Verticillium* in melon and cotton plants [58,59].

Zargar et al. [60] found that the antifungal activity of the endophytic *Penicillium* spp. occurring in natural strawberry flora was effective in controlling the anthracnose disease caused by *Colletotrichum nymphaeae* and alleviated its harmful effects versus the control significantly. Bhuiyan et al. [61] proved the potential capability of *Penicillium* sp. isolates in suppressing the causal agent of sugary disease of sorghum, i.e., *Claviceps africana*. On the other hand, Doley et al. [20] revealed that the treatment of peanut plants with mycorrhiza and *T. viride* was effective in stimulating growth and increasing the nutrient absorption area of the plant. The fungi also reduced the damage of *S. rolfsii* efficiently. These results support our findings and highlight the utility of endophytes in *S. rolfsii* biocontrol.

## 5. Conclusions

The general results of the research performed in vitro and in vivo revealed that the peanut stem white rot disease can significantly be controlled by the antagonistic fungi occurring in peanut natural flora, including *T. viride*, *A. flavus*, and *P. rubens*. Based on the results, *T. viride*, *A. flavus*, and *P. rubens* (MN395854.1) were the most effective isolates in suppressing the pathogen and improving *A. hypogea* growth indices in both in vitro and greenhouse conditions. After conducting trials in field conditions in future studies, this research can be promising in applying endophytic agents as an important part of the integrated management of this severe disease.

## Figures and Tables

**Table 1:** Sequence of primers used in the PCR.

Name	Sequence (5′→3′)	TM	GC%	Mer
18s rDNA-F	CACCAGACTTGCCCTCCA	58.24	61.1	18
18s rDNA-R	AACCTGGTTGATCCTGCCAG	59.35	55	20

TM: melting temperature; GC%: GC Content; Mer: number of nucleotides in each primer.

**Table 2:** Effect of antagonistic fungi on the mycelium growth inhibition of *Sclerotium rolfsii* in the dual culture, volatile metabolites, and non-volatile metabolites methods.

Treatments	Dual Culture (%)	Volatile Metabolites (%)	Non-Volatile Metabolites (%)
*Aspergillus flavus*	45.50 ± 0.01 ^c^	73.50 ± 0.08 ^b^	0.00 ± 0.00 ^c^
*Penicillium decaturense*	0.00 ± 0.00 ^d^	0.00 ± 0.00 ^d^	0.00 ± 0.00 ^c^
*Penicillium rubens* (MN395854.1)	65.15 ± 0.02 ^b^	84.30 ± 0.01 ^a^	90.20 ± 0.10 ^b^
*Penicillium rubens* (MN395851.1)	0.00 ± 0.00 ^d^	7.03 ± 0.00 ^c^	0.00 ± 0.00 ^c^
*Trichoderma virens*	90.98 ± 0.15 ^a^	0.00 ± 0.00 ^d^	0.00 ± 0.00 ^c^
*Trichoderma viride*	66.89 ± 0.11 ^b^	0.00 ± 0.00 ^d^	91.80 ± 0.01 ^a^

Treatments having at least one similar letter do not show a significant difference according to the LSD test at *p* ≤ 0.05.

**Table 3:** Effect of antagonistic fungi on the disease severity, reduction in disease severity, and incidence of peanut white stem rot disease under greenhouse conditions.

Treatment	Disease Rating ± SE	Reduced Severity of Disease (%) ± SE	Disease Incidence (%) ± SE
*Aspergillus flavus +* *S.* *rolfsii*	2.90 ± 0.09 ^cd^	42.00 ± 0.01 ^b^	32.00 ± 0.09 ^d^
*Penicillium decaturense +* *S.* *rolfsii*	3.30 ± 0.44 ^bc^	32.00 ± 0.07 ^c^	38.00 ± 0.02 ^cd^
*Penicillium rubens* (MN395851.1) *+* *S.* *rolfsii*	3.40 ± 0.50 ^bc^	34.00 ± 0.10 ^c^	44.00 ± 0.12 ^c^
*Penicillium rubens* (MN395854.1) *+* *S.* *rolfsii*	3.06 ± 0.38 ^c^	38.00 ± 0.10 ^bc^	50.00 ± 0.15 ^b^
*Trichoderma virens +* *S.* *rolfsii*	3.60 ± 0.44 ^b^	28.00 ± 0.08 ^d^	52.00 ± 0.14 ^b^
*Trichoderma viride +* *S.* *rolfsii*	2.80 ± 0.44 ^d^	44.00 ± 0.06 ^b^	31.00 ± 0.04 ^d^
Antagonistic fungi (only)	0.00 ± 0.00 ^e^	100.00 ± 0.00 ^a^	0.00 ± 0.00 ^e^
Distilled water (negative control)	0.00 ± 0.00 ^e^	100.00 ± 0.00 ^a^	0.00 ± 0.00 ^e^
*Scierotium rolfsii* (positive control)	5.00 ± 0.01 ^a^	00.00± 0.00 ^e^	100.00 ± 0.00 ^a^

Treatments in columns having at least one similar letter did not show a significant difference according to the LSD test at *p* ≤ 0.05.

**Table 4:** Effect of antagonistic fungi on the height, fresh weight, and dry weight of peanut plants infected and non-infected with *Sclerotium rolfsii*.

Treatments	Height with Root (cm) ± SE	Fresh Weight with Root (g) ± SE	Dry Weight with Root (g) ± SE
*Aspergillus flavus +* *S.* *rolfsii*	64.33 ± 1.76 ^ab^	14.17 ± 1.43 ^b^	2.19 ± 0.03 ^b^
*Aspergillus flavus* (only)	60.33 ± 0.33 ^b^	10.24 ± 0.76 ^bc^	1.21 ± 0.08 ^cd^
*Penicillium decaturense +* *S.* *rolfsii*	63.33 ± 0.67 ^ab^	11.30 ± 1.11 ^bc^	3.33 ± 0.73 ^a^
*Penicillium decaturense* (only)	60.33 ± 0.33 ^b^	6.52 ± 2.22 ^cd^	2.23 ± 0.34 ^b^
*Penicillium rubens* (MN395851.1) *+* *S.* *rolfsii*	63.33 ± 1.45 ^ab^	7.15 ± 2.21 ^c^	2.03 ± 0.49 ^bc^
*Penicillium rubens* (MN395851.1) (only)	60.67 ± 0.67 ^b^	6.05 ± 1.74 ^cd^	1.67 ± 0.37 ^c^
*Penicillium rubens* (MN395854.1) *+* *S.* *rolfsii*	67.00 ± 0.14 ^a^	6.46 ± 1.21 ^cd^	2.33 ± 0.23 ^b^
*Penicillium rubens* (MN395854.1) (only)	61.00 ± 1.00 ^b^	5.57 ± 0.88 ^d^	1.48 ± 0.18 ^c^
*Trichoderma virens +* *S.* *rolfsii*	60.33 ± 0.33 ^b^	4.52 ± 0.43 ^e^	1.82 ± 0.46 ^c^
*Trichoderma virens* (only)	60.00 ± 0.00 ^b^	2.76 ± 1.09 ^f^	1.16 ± 0.01 ^cd^
*Trichoderma viride +* *S.* *rolfsii*	68.00 ± 1.11 ^a^	17.26 ± 0.26 ^a^	1.88 ± 0.21 ^c^
*Trichoderma viride* (only)	65.33 ± 1.45 ^ab^	12.00 ± 0.00 ^bc^	1.62 ± 0.26 ^c^
Distilled Water (negative control)	60.03 ± 0.77 ^b^	6.06 ± 0.77 ^cd^	1.35 ± 0.17 ^cd^
*Sclerotium rolfsii* (positive control)	33.55 ± 0.36 ^c^	2.18 ± 0.13 ^f^	1.00 ± 0.11 ^d^

Treatments in columns having at least one similar letter did not show a significant difference according to the LSD test at *p* ≤ 0.05.

## Data Availability

Not applicable.
